# A New Idea for the Spread of the Knowledge of Medical Ethics

**Published:** 1883-04

**Authors:** 


A New Idea for the Spread of the Knowledge of Medical Ethics.—The code of medical ethics contains much that relates exclusively to "the duties of patients to their physicians." It has, doubtless, puzzled many to understand how patients were to be made aware of their duties to their physicians. Clearly the code has never been brought to public notice. All the public know of medical ethics has been derived from physicians and newspapers. As to the desirability of instructing the people respecting their duties in this regard, there can be no doubt. As to the best method for accomplishing it, there is plenty of room for ingenuity. Dr. F. H. Darby, of Morrow, O., has struck upon the idea of using wrapping paper as the means of spreading this knowledge. Thus he has had printed on one side of wrapping paper the duties of patients to physicians, as given by the code. This printing is so arranged that the sheet can be torn or cut into halves, quarters, eighths, or sixteenths, without destroying the reading matter. There are also added items of general interest, and of wit and wisdom, to still farther attract attention. This paper is for the use of physicians and druggists in dispensing their medicines. If properly carried out, we have no doubt but this scheme will be productive of general good. Perhaps it may be the means of stimulating other minds to devise other and better modes for the instruction of people in these and other important matters relating to their relation to the medical profession.

Thus it may be hoped that some of the heresy and skepticism now existing among the people may be removed.—*Detroit Lancet.*

